# A dendrogram approach to the structure of spike trains

**DOI:** 10.1186/1471-2202-12-S1-P153

**Published:** 2011-07-18

**Authors:** Conor Houghton

**Affiliations:** 1 School of Mathematics, Trinity College Dublin, Dublin, Ireland

## 

A novel mathematical description for the temporal structure of spike trains is presented. This works by mapping the spike train to a dendrogram produced by hierarchical clustering. The branch-length structure of the dendrogram is equivalent to the distribution of inter-spike intervals, but morphological descriptions of the dendrogram can be used to quantify other aspects of the temporal structure of spike trains. In this way, it is shown that example sets of spike trains have more structure than is accounted for by the distribution of inter-spike intervals. The goal is to segregate the loose notion of “sparseness” into two different aspects of temporal structure, the distribution of inter-spike intervals and the morphology of the dendrogram constructed during clustering and to provide a tool for analyzing and comparing spike train properties.

**Figure 1:**
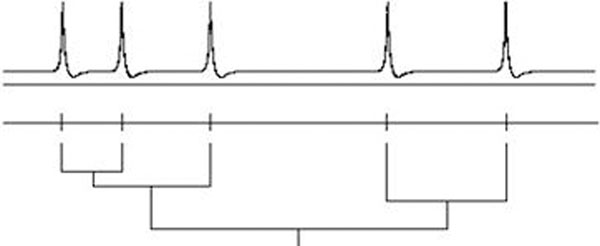
The spikes are clustered at different time scales to give an agglomerative dendrogram.

Roughly, the distribution of inter-spike intervals fails to describe the temporal granularity caused by the clustering of spikes. The difficulty with quantifying this is in deciding how close two spikes need to be for them to be agglomerated in clusters. The approach taken here is to avoid that choice by performing a hierarchical clustering: the spikes are agglomerated into larger and larger groups as a timescale is increased, this is illustrated in Figure 1. Quantifying morphological properties like the bushiness of the resulting dendrogram then describes the temporal structure of spike trains. In fact, hierarchical clustering is of interest to computer scientists and this provides a number of candidate methods for quantifying the morphology of dendrograms, the one used here was introduced in [1] and scores dendrograms from zero, for the leggiest, to one, for the bushiest.

This quantification is applied to an example spike-train data set described in [2] and made available on http://neurodatabase.org. The data consist of single cell recordings from the nucleus of the solitary tract in rat during presentation of taste stimuli. With a clear separation between the stimulus timescale and the temporal scale of spiking, these data are chosen to present a kind of worst-case scenario for the proposal. Nonetheless, when the real data is compared to artificial data produced by shuffling the inter-spike intervals, the Mann–Whitney U test shows that real data has a significantly higher value of the morphological parameter introduced here.
